# Complex Concentrated Alloys for Substitution of Critical Raw Materials in Applications for Extreme Conditions

**DOI:** 10.3390/ma14051197

**Published:** 2021-03-04

**Authors:** Dumitru Mitrica, Ioana Cristina Badea, Beatrice Adriana Serban, Mihai Tudor Olaru, Denisa Vonica, Marian Burada, Radu-Robert Piticescu, Vladimir V. Popov

**Affiliations:** 1 National R&D Institute for Nonferrous and Rare Metals-IMNR, 077145 Ilfov, Romania; dmitrica@imnr.ro (D.M.); beatrice.carlan@imnr.ro (B.A.S.); o.mihai@imnr.ro (M.T.O.); denisav22@gmail.com (D.V.); mburada@imnr.ro (M.B.); rpiticescu@imnr.ro (R.-R.P.); 2 Israel Institute of Metals, Technion R&D Foundation, Haifa 3200003, Israel; vvp@technion.ac.il

**Keywords:** critical raw materials, non-ferrous alloys, CCA, modeling

## Abstract

The paper is proposing a mini-review on the capability of the new complex concentrated alloys (CCAs) to substitute or reduce the use of critical raw materials in applications for extreme conditions. Aspects regarding the regulations and expectations formulated by the European Union in the most recent reports on the critical raw materials were presented concisely. A general evaluation was performed on the CCAs concept and the research directions. The advantages of using critical metals for particular applications were presented to acknowledge the difficulty in the substitution of such elements with other materials. In order to establish the level of involvement of CCAs in the reduction of critical metal in extreme environment applications, a presentation was made of the previous achievements in the field and the potential for the reduction of critical metal content through the use of multi-component compositions.

## 1. Introduction

First debates about supply risks date back to the late 1930s, but in the last 10 years, a great concern has arisen about supply security of strategic elements, especially from import-dependent industrialized countries whose high-tech products are strongly dependent from them. Since 2010, EU released a list of strategic elements and materials, the so-called critical raw materials (CRMs) list, which is updated every three years to take into account the evolving scenarios of demand (economic importance) and supply risks of the critical elements. Table 1 shows the last CRMs list, released in 2020 [1], in which the new entries for CRMs are marked in bold.

It is noticeable, how the deployment of renewable energy generation and e-mobility solutions has translated into raw materials demands, leading to “new entries” such as lithium, which was never included in the previous lists. Moreover, many of the raw materials assessed in the 2020 list are also essential for the development of other strategic sectors such as defense and aerospace, robotics and digital technologies, and additive manufacturing.

Several efforts have been made for the partial or total substitution or recycling of CRMs for various applications, but serious difficulties appear when the usage of known critical materials is correlated with the basic functionality or required performance of the final device. Thus, gallium and indium are necessary for light-emitting diode (LED) technology; silicon is needed in semiconductors; lithium represents the main ingredient in today’s batteries; permanent magnets based on rare earth metals have over 10 times higher magnetization capacity; tungsten is required for high-temperature stability; the magnesium found in light alloys is known for its high strength, low density, and corrosion resistance; and cobalt is used frequently in magnetic materials, batteries, and superalloys. However, even the task for complete substitution of critical materials in these applications is almost unobtainable, reduction of critical material content for industrial use or improved recycling technologies represent realistic solutions.

Conventionally, metallic alloys are comprised of a major principal element and other minor elements added to improve certain properties. The new concept of high entropy alloys (HEAs) is based on the original theory that assumes that a large number of principal elements would induce a large configurational entropy and attract the formation of predominant and easier to control solid solution structures [2]. Multi-principal element alloys (MPEA) [3] and HEAs [2], which were originally discovered as new types of alloys almost 17 years ago, have evolved recently in a more realistic definition as compositionally complex alloys [4] or complex concentrated alloys (CCAs) [5]. The new definitions were stipulated due to the uncertainty in the role of entropy on the stability of the multi-principal alloys (MPA). Several authors [6,7,8] studied the thermodynamic criteria for the formation of solid solution structures in multi-component alloys, and other parameters such as formation enthalpy or atomic radius difference, seem to have a higher influence on the type of the alloy structure. The translation from compositionally complex alloys to complex concentrated alloys was realized within one year of publications by the same authors [4,5]. More likely, the reason behind this was that compositionally complex alloys’ expression refers to alloys that have a complex composition, with a large number of elements but not necessarily in a large proportion for each element. On the other hand, complex concentrated alloys’ definition suggests that a higher concentration of component elements is required. In this review, the authors used the most recent definition as complex concentrated alloys. Table 2 shows the main definitions related to the subject of the current review.

The review contains the most recent achievements in the critical metal strategy and CCA development. In order to present the capability of CCAs for the substitution or reduction in the use of various critical metals, a principal description of the current state of the art in CRMs policies, CCAs design strategies, and applications is necessary. In conclusion, the presentation contains three main chapters that discuss the following issues:

- European critical agenda: shows the most recent strategy in dealing with critical raw material resilience. Future application trends are also presented;
- CCA family and research trends: a general presentation of CCAs including the research work for high entropy alloy concept. The application-oriented research is also reviewed in this chapter;
- Critical raw material substitution challenges and CCA potential: the capability of CCAs to aid in the process of CRMs reduction, with specific reference to the applications in extreme conditions, is presented here in detail. The potential and future trends in alloy development are also covered by this chapter.

## 2. European Critical Materials Agenda

Due to the extended usage of several materials in today’s demanding economy, the natural resources for providing such raw materials became more and more important to determine the adoption of dedicated studies and strategies for avoiding a crisis shortage. In this respect, the European Union has issued several reports on the critical materials at least every 3 years.

**Figure 1:**
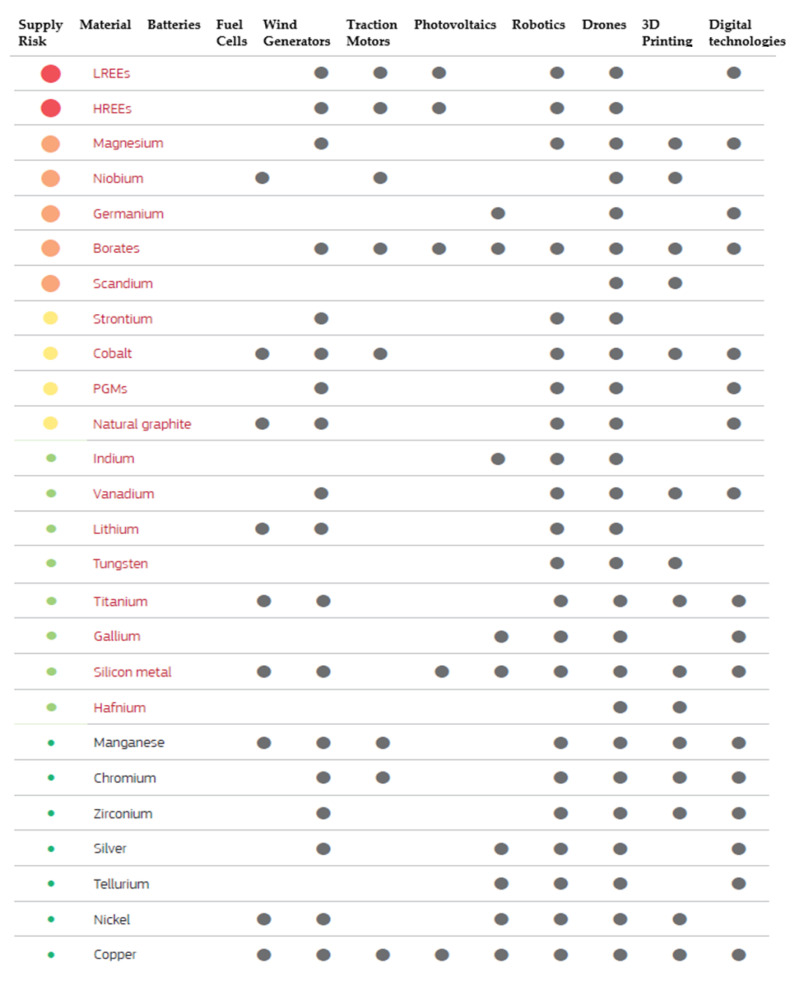
Critical and non-critical raw materials used in different technologies (selected top 25 materials) [9].

**Figure 2:**
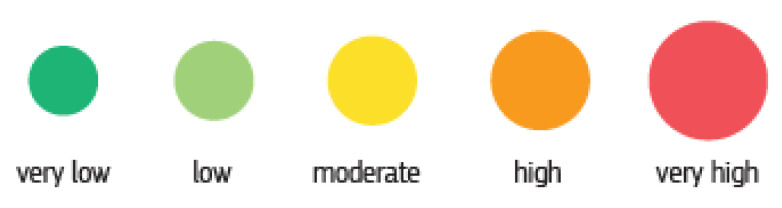
Supply risk indication [9].

With the continuous advancement of technology and the need more than ever for alternative energy sources for a sustainable economy, the world has promoted a great interest in the development of more and efficient electrical vehicles (Li-ion batteries), more renewable energy supply (eolian, photovoltaic, fuel cells) or advanced industrial technologies (robotics, drones, 3D printing). Each of these industrial fields is based on an important supply of raw materials, which needs to be addressed before it becomes a critical shortage. A selection of critical and non-critical raw materials used in these technologies are shown in Figure 1. The analysis bottlenecks for each technology can result in a very low supply risk and very high supply risk, based on market research reports and publicly available information (Figure 2) [9].

Critical raw materials are distinguished from non-critical raw materials by being written in red. Moreover, light rare earth elements (LREEs), heavy rare earth elements (HREEs), and platinum group metals (PGMs) are groups of multiple raw materials.

The European Commission treats the critical raw materials topic as an area where Europe needs to act urgently to prepare future stocks and develop resource autonomy by reducing import dependence, enhance the circular economy and be resource-efficient. The EU plan to resolve the dependency issue is based on developing resilient value chains for industrial ecosystems, enhancing the circular use of resources, and developing sustainable and innovative products [1]. The most important supply chains that have high risks at different levels include electric vehicles, batteries and energy storage, and rare earth for permanent magnets.

According to the latest reports [1,10], which sets bases for defining the CRMs in Europe, their distribution, supply shortage, and major utilization, the European Commission has identified a list of 30 economically important raw materials that are subject to a higher risk of supply interruption, i.e., antimony, baryte, beryllium, bismuth, borate, cobalt, coking coal, fluorspar, gallium, germanium, hafnium, heavy rare earth elements, light rare earth elements, indium, magnesium, natural graphite, natural Rubber, niobium, platinum group metals, phosphate rock, phosphorus, scandium, silicon metal, tantalum, tungsten, vanadium, bauxite, lithium, titanium, and strontium.

A large number of metals are contained in this group of critical materials. Among them are found metals used frequently in applications of extreme conditions: Co, Hf, Nb, PGMs, Ta, Ti, V, and W. Most of these elements have a good combination of several required properties, namely, high mechanical resistance, high melting point, and good corrosion resistance.

Aerospace is one of the strategically important fields in which almost all technological developments are included due to its complexity of systems and materials used [9,11]. For example, robots play an increasing role in the future of manufacturing, in which emerging technologies such as automated metal processing routs, automated assembling, and testing conveyors gain more trust and interest. Additive manufacturing will soon reshape and replace conventional manufacturing of parts [12,13]. This will lead to changes in the number of materials used and types of raw materials. Lightweight parts with complex geometrics are the key to 3D printing along with production in remote locations that supports strategic and tactical planning.

The aerospace industry needs to overcome the biggest challenges because it uses a number of very specialized and complex structured materials such as aluminum alloys, steel alloys, titanium alloys, magnesium alloys, cobalt- and nickel-based super-alloys, ceramics, composites, etc. With the progress of this industry, the new generations of aircraft replaced almost 50% of materials used with composites. Traditional materials are being replaced with new lightweight materials based on titanium alloys, composites, and high-temperature resistant plastics. This can be translated into higher maneuverability, higher accuracy, and higher autonomy of airplanes.

Among the refractory elements, tungsten has the highest melting point and was mostly used in the past, in filament light bulbs. Over time the consumption decreased due to the appearance of more efficient fluorescent and led technologies [14]. Nowadays, tungsten is used mostly at the fabrication of hard cutting tools (as carbide) and arc-welding electrodes. Tungsten carbide hardness is second to diamond and can be produced relatively easily through a well-known process.

The platinum group materials (PGMs) and especially platinum and palladium provide highly effective operation of various conventional devices in computer hardware, robotics, and medicine. The criticality of PGMs is mainly influenced by the projected development of fuel cell production, especially for the automotive industry, where these metals provide the most efficient catalysts. Recent developments showed partial substitution (up to 20% Pt) with Co and other metals with a compromised decrease in functionality [9].

The remaining refractory metals—Hf, Nb, and Ta—have various usage (electrical components, nuclear control rods, medical implants, jet engine parts, and chemical plant components) generated by their special properties: high hardness, high melting temperature, solid solution strengthening effect, superconductivity, corrosion resistance, and biocompatibility. Partial substitution of these elements in destinated applications is possible only at expense of lower performance.

## 3. Complex Concentrated Alloy Families and Research Trends

The recently discovered CCAs are multi-component advanced metallic materials that promise a wide range of mechanical and physical properties, such as high strength and toughness, high stiffness, and improved oxidation resistance. CCAs are a mixture of four or more elements in high proportion. The high mixing entropy of CCAs generates the potential for solid-solution simplified microstructures. The multi-component nature of CCAs, the sluggish diffusion, and severe lattice distortions have a significant effect on their capability to maintain high mechanical properties at high temperatures [15].

The properties of the alloys used for various applications depend to a large extent on their composition. By replacing one or more elements in the composition of the CCAs, significantly different properties can be obtained from the initial ones. Moreover, decreasing or increasing the number of alloying elements can generate different structures with important influences on the properties of the alloys [16].

Due to the large number of possible combinations of metals that can be used for CCA synthesis, a number of selection criteria have been defined for metals that can be used for materials with certain properties.

**Figure 3:**
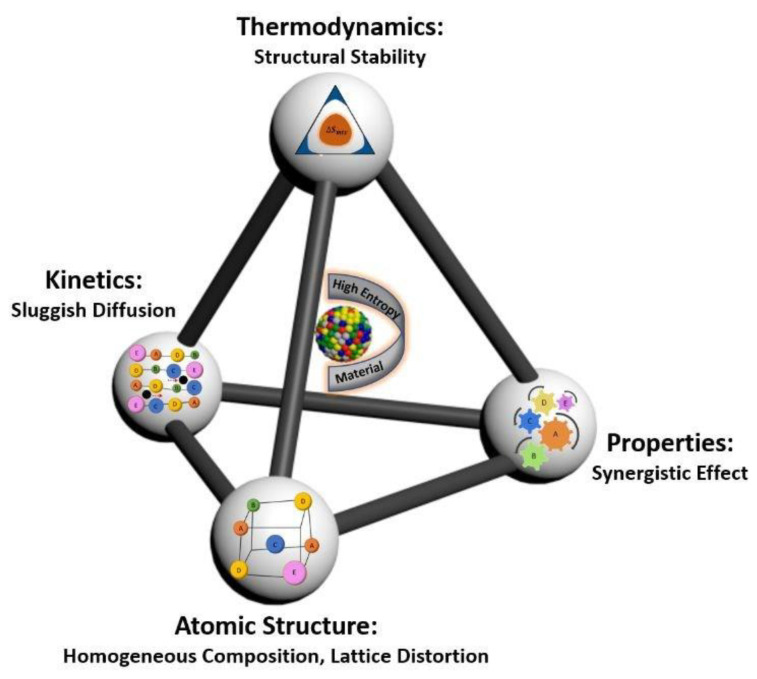
The four core effects of CCAs [17].

To design suitable alloys that are able to reduce the amount of CRMs, it is important to have in view the four core effects of CCAs because of the diversity of the multiple elements that are alloyed to obtain specific properties (Figure 3) [17].

In order to determine the capacity of the alloy systems to form CCAs containing structures formed of stable solid solutions, the thermodynamic evaluation was performed by the theoretical criteria established in the literature [18]. The principle underlying the existence of CCAs is that by increasing the number of main alloying elements, solid solutions are preferentially formed. This principle derives from Boltzmann hypothesis on the connection between entropy and the number of elements in the system, which means that for an almost equimolar alloy, with at least five main alloying elements, the change in configurational entropy during solid-state formation is greater than the necessary formation of intermetallic compounds [19].

In addition to the entropy criterion, the composition of the CCAs should meet other conditions. The mixing enthalpy (ΔH_mix_) has an important contribution to the formation of intermetallic secondary phases in the final structure of the alloy. Therefore, solid solution formation criteria have been studied by several authors [7], associating phase formation criteria with the Hume-Rothery rule, in which small differences in atomic radius (δ), electronegativity (Δx), and electrons valence play an important role in the selection of the alloys. Later, Yang and Zhang [8] introduced the factor Ω to express the influence of mixing enthalpy on the formation of solid solution phases. Due to the contradictory experimental results obtained so far, the theoretical selection criteria have been further developed by other authors with particular emphasis on the formation of intermetallic phases and less on the degree of stability of solid solutions. Therefore, it was established that content higher than 40% of the coefficient of the sigma-forming element (PSFE) has an important role in the formation of sigma phases in CCAs [20]. Another criterion for determining the presence of intermetallic phases in HEAs was recently provided by Senkov and Miracle [21,22]. The authors formulated a k_1cr_ factor based on annealing temperature, mixing entropy, and mixing enthalpy, which must be greater than the ratio between the enthalpies of intermetallic compound formation and solid solution formation, to form preponderantly solid solutions.

Due to the structural complexity of the CCAs, it is necessary to use computational modeling techniques to obtain the necessary properties to be used in extreme conditions. The modeling process includes the usage of the CALPHAD (CALculation of PHAse Diagrams) method, to realize kinetic and thermodynamic calculations, which are useful in quantizing the Gibbs free energy of the constituent phases of the system [23,24,25]. A method used to analyze the multi-component alloy systems is called density functional theory (DFT), which requires introducing data regarding the atomic numbers to obtain the electronic properties of the solids. In order to predict the behavior of an alloy through ab-initio molecular dynamics (AIMD) simulations, it is not necessary to provide information regarding the empirical interatomic potential. Another instrument used to research material dynamics in atomistic scale is Monte-Carlo (MC), which is similar to the AIMD method and is based on the potentials of Chen’s lattice inversion pair functions. The modeling results demonstrated that CCAs with body-centered cubic (BCC) structures have greater distortions than face-centered cubic (FCC) CCAs. A new approach in computational modeling is considered the Lederer-Toher-Vecchio-Curtarolo (LTVC) method, which incorporates data regarding energies into a statistical mechanical model, in order to predict more exactly the temperature of transition from a multi-component system to a solid solution phase [26].

**Figure 4:**
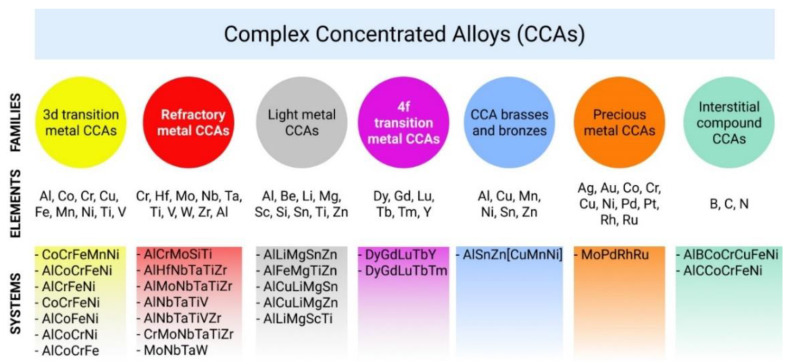
The seven CCA families illustrated by element groupings as defined in [18].

The present and future applications strategy of CCAs is best explained by Miracle and Senkov in [18]. Figure 4 shows the proposed classification of the seven main CCAs families.

The first includes systems built using the so-called transition metals (3D alloys)—aluminum, cobalt, chromium, copper, iron, manganese, nickel, titanium, and vanadium. This CCA family is the most representative and widely researched. There is a large volume of research performed on alloys containing Fe, Cr, Co, Ni, Cu, or Ti compared with well-known commercial alloys (stainless steels and superalloys).

Because the high entropy alloy concept was developed mostly around transitional elements, which seem to combine very well in equal or near-equal multi-component proportion, the natural option in the development of the new alloys were high strength and high-temperature applications.

The second family is also big and represents refractory CCAs [27]. The systems of the second CCA family use chromium, hafnium, molybdenum, niobium, tantalum, titanium, vanadium, tungsten, zirconium, and aluminum. High-temperature stability mainly required in turbine blade manufacturing was put to the test with HEAs containing Ta, Mo, W, Nb, and Hf, and promising results have been obtained [28,29,30,31,32]. Low-density metals such as titanium and aluminum are used to decrease the weight of these CCAs and to substitute refractory more expensive elements of this family [28,31,32].

The third light metal CCA family using aluminum, beryllium, lithium, magnesium, scandium, silicon, tin, and zirconium, has a goal to design lightweight materials with high structural performance [33,34].

The fourth of CCA families using dysprosium, gadolinium, lutetium, terbium, thulium, and yttrium is investigated with the focus on synthesizing a single-phase hexagonal closest packed (HCP) olid solution [34].

For the fifth family–CCA brasses and bronzes—the main motivation is to increase the machinability and strength of hese materials [18,35].

The sixth noble metal CCAs are aimed to reduce alloy cost by substituting the most expensive palladium and platinum with less expensive gold and ruthenium, and even much cheaper cobalt, chromium, copper, and nickel. This is extremely relevant for these modern applications as in a catalytic industry and new jewelry design [34].

According to the proposed terminology, the new CCA families presented in Figure 4 are the developing ones and less represented in literature than the first two families [18].

In Figure 4, the used elements for each CCA family are presented. Moreover, we illustrated some examples of the most investigated CCA chemical compositions concerning each family.

**Figure 5:**
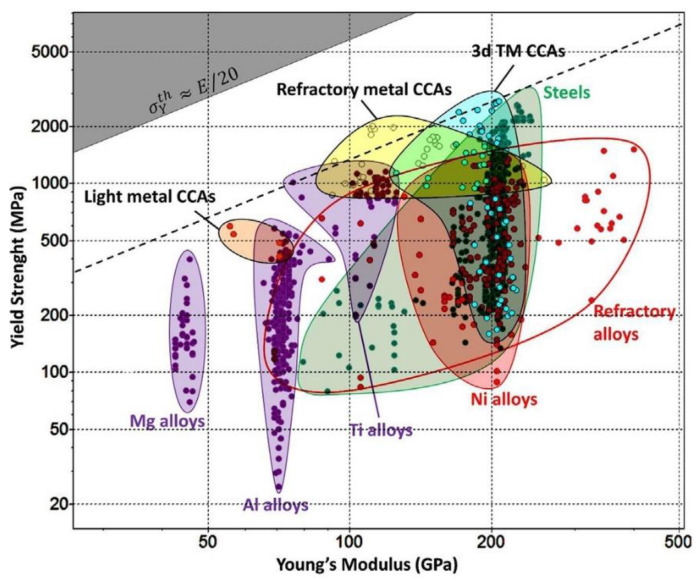
Yield Strength vs. Young Modulus for conventional metal alloys and CCAs [5].

The diagram presented in Figure 5 shows a comparison between the family of CCAs and conventional alloys (Mg-, Al-, Ti-, Fe- and Ni-based alloys and refractory alloys). Each family of alloys occupies a particular area of property space, represented by large and colored bubbles. The upper left gray corner shows the theoretical strength (σ_y_ = E/20) delimiting the boundary of the inaccessible region of the plot. CCA classes are among the wide range of conventional alloys considered. It can be seen that light metal CCAs are found in the space between the Mg alloys class and Al alloys class, which offers new design options for this new class of materials. In contrast, refractory metal CCAs are located at the top, closer to the theoretical tight yield limit than other alloy families [5].

Part of the material property space from 3d TM CCAs covers a narrow space between the Ti, Ni alloys class, and steel class, and it is very close to the theoretical limit of yield strength. High values of yield strength can be related to the microstructure of these types of alloys. Thus, CCAs can be considered as a new class of materials that can compete or even replace conventional alloys [5].

The large number of results provided by numerous researchers in studying actual and potential properties of HEAs, and more recently of the CCA families, has directed the research mainly toward high-temperature, multi-functional, and structural applications. 

The concept of CCAs and freedom of choice in alloy development, due to the large number of elements, allows for great flexibility in the choice of properties and applications. In the same alloy system, the increasing or decreasing proportion of one element can have a significant impact on the microstructure and properties. Therefore, the multi-component alloys design concept can provide interesting applications, which are dependable on materials with high critical element content [18]. A promising application of multi-component alloys is hydrogen accumulation as a fuel for electricity and transportation industries, to obtain reversible hydrogen storage [36]. Hydrogen can be considered an attractive alternative of decreasing fossil fuel demands and reduce the negative environmental impact of the compounds resulted in the combustion processes [37].

Another category of multi-component alloys application is represented by shape memory alloys. They are characterized by shape memory effects, which make these alloys suitable to be used in different fields, such as pseudoelastic, robotic, aerospace, thermal, or mechanical areas [38].

Materials with thermoelectric properties are a category of multi-component alloys with numerous applications in converter devices that serve for the direct transformation of heat in electricity [39].

To obtain the necessary properties to be operated in special conditions, the multi-component alloys have in their chemical composition critical metals. Although the European Commission is trying to reduce the usage of CRMs, they are very difficult to replace due to their specific properties. A possibility to reduce or replace them, where possible, is to design and elaborate CCAs, based on non-critical raw materials, which, in combination with other elements, give the alloy the properties needed to be used in various applications, such as those in extreme conditions.

## 4. Critical Raw Material Substitution Challenges and CCA Potential

**Figure 6:**
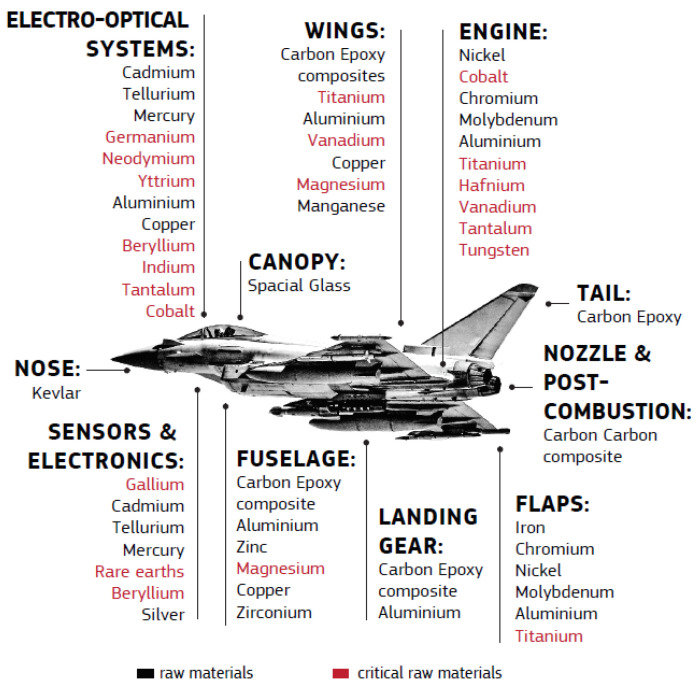
Materials distribution in different parts of the combat aircraft [9].

Many engineering applications require materials that have as their main condition the ability to be used in extreme conditions of temperature, pressure, or corrosion. Materials that work in these harsh environments must maintain their performance at high levels. For example, turbine blades in jet engines operate very close to their melting temperature in an oxidizing environment [40]. The materials used in different parts of the fighter aircraft are presented in Figure 6 [9]. The elements from the CRM group are written in red.

In order to understand why these critical materials are important elements in the aerospace sector and what are the challenges in their substitution or quantitative reduction, it is important to analyze each element individually.

Cobalt is a material of great interest in aircraft engine production due to its resistance to high temperatures. In general, Co is used as an alloying element, so that Co-based alloys make a difference through properties such as wear resistance, creep resistance and fatigue at high temperatures, and sulfidation resistance. In addition to the crystallographic nature of cobalt, the alloying elements influence these properties by the formation of solid solutions with Cr, W, and Mo, binder for metallic carbides, and high corrosion resistance due to the alloying with Cr. Moreover, Co-based alloys that have an increased oxidation resistance are used for high-temperature applications, such as gas turbine blades and buckets. Moreover, a high Cr content in Co alloys provides superior corrosion resistance in extreme conditions and resistance to thermal fatigue and weldability, superior to Ni-based alloys [41]. The corrosion resistance of Co-alloys can also be significantly improved by alloying with Al because Al has an advantage over Cr, i.e., it forms a thin and stable Al_2_O_3_ scale at high temperatures [42]. The Cr_2_O_3_ scale, although initially protective, is prone to degradation (so-called chromia evaporation) during long-term exposures in oxidizing atmospheres at high temperatures.

Titanium is mostly used in combination with iron, vanadium, molybdenum to produce strong and light alloys for the aerospace industry. Due to the high tensile resistance/mass ratio, high corrosion resistance, fatigue, cracking, and the ability to withstand relatively high temperatures without losing their properties, titanium alloys are used in the aerospace industry. Basically, about two-thirds of the titanium produced is used in engines and structural elements for aircraft. The Ti_6_Al_4_Valloy represents about 50% of the consumption of alloys in the aeronautical industry [11,43].

TiAl alloy is used for motor applications due to its properties of operating in high-temperature conditions [44,45]. Ti aluminides, which are part of Ti-based alloys, have been studied extensively in recent years but have been introduced into production for ductility reasons since eight years ago. Although Ti-based alloys have special advantages over other materials, they are some of the most expensive materials used today in airframes. The cost of Ti alloys becomes lower because they can be replaced, for example, with CCAs.

In the aerospace industry, vanadium also plays an important role because it provides low density and hence the ability to maintain a high resistance to operation at high temperatures. These are the essential conditions for the materials used in the engine gas turbines. Vanadium alloying gives the alloy creep resistance at temperatures up to 550 °C. For example, aerodynamic gas turbine bearings are often made of steel containing 1% vanadium with 18% tungsten and 4% chromium [46]. Furthermore, vanadium is used as an alloying product in Ti alloys for the aerospace industry [47].

Other elements used in the aeronautical industry, which are found in the list of CRMs are tantalum and hafnium. Hafnium’s properties of stability and resistance to extreme temperatures, both in metallic and compound form, make it a key element in aeronautical applications [48].

The concern about Ta carbide (TaC) and Hf carbide (HfC) increased in recent years due to their extremely high melting temperature, hardness, and high elastic modulus, in addition to their ability to form solid solutions [49].

The use of Hf in Ni-based alloys helps strengthen grain boundaries, improving the creep at high temperatures and the tensile strength. In addition, with its high affinity for carbon, nitrogen, and oxygen, the metal also provides hardening by dispersing particles in the second phase [48].

In inert atmospheres, tantalum and its alloys have good mechanical properties at extreme temperatures. Tantalum added to Ni-based superalloys gives them important mechanical properties at higher temperatures, better resistance to hot corrosion, and longer life. Tantalum forms solid solutions with other refractory metals, such as molybdenum, tungsten, and niobium [50,51].

Turbines used in aeronautical jet engines have highly demanding requirements regarding the component materials. The high temperatures and corrosive gases produced in the combustion chambers represent extreme conditions for the working parts in the engine hot sections.

Presently, Ni-based superalloys are the most used materials for the manufacturing of main components in gas engine turbines due to the combination of properties that are developing—creep resistance, temperature resistance, environmental resistance, and damage tolerance. The high properties of Ni superalloys are derived mainly from the dispersion of Ni-Al precipitates in the alloy mass. There is substantial research on Ni-based superalloys that have reached a critical point due to the softening mechanism that occurs at high temperatures [29].

Considering the properties that a material operating in extreme conditions must fulfill, the CCAs are the most suitable. These alloys differ from traditional alloys especially by having a large number of elements and a complex structure that allows for the presence of intermetallic phases, with good stability at elevated temperatures [52].

The potential of CCAs to replace conventional alloys with a high content of critical material is provided by the high number of components and the large spectrum of compositional variations that can be applied to obtain specific properties. Partial or total critical element substitution in conventional alloys would be possible by large alloy configurations.

In order to be a viable solution, the developed CCAs for extreme environment applications, such as jet engines, should have high operating temperatures of above 700 °C, withstand thousands of hours of operation in extreme environments, a high value of the gas flow resistance (300–400 MPa) to withstand the tensile stresses, and high creep resistance properties of over 1200 h at 1200 MPa. Other important characteristics that CCAs used in extreme conditions should meet are low density (less than 8 g/cm^3^) and high resistance to fatigue to withstand the numerous cycles of variable rotation stresses to minimize the power required to drive the compressor and move the entire weight of the engine. Materials should have oxidation resistance and good resistance to chemical corrosion and cavitation erosion caused by flue gases rich in carbon, nitrogen, and Sulphur [53,54].

Alloys currently used in the hot area of a jet engine are mainly based on a high content of Ni and Co (Inconel 617, Inconel 718, Inconel 792, Hastelloy x, Haynes 230, Triballoy 800, MAR-M-247, etc.). Ni-based superalloys have the advantage of forming intermetallic γ’ precipitates that improve considerably the mechanical resistance and hot hardness of the alloy. Co-based alloys are also known to provide increased hardness and good corrosion resistance at high temperatures through the solid solution strengthening effect. The causes for the popularity of nickel-based alloys are excellent mechanical strength, good hot and cold machinability, the best weldability of all superalloys, and moderate cost.

The influence of alloying elements on the physical, mechanical, and chemical properties of CCAs is significant and is an important factor in obtaining an optimal material for applications in extreme conditions. Fe and Ni are found in most of the CCAs due to their ability to form thermodynamically stable alloys in combination with other elements. Cr has the role of increasing the mechanical strength and corrosion resistance, and it improves the casting properties and oxidation resistance. Co improves the stability of structures based on complex solid solutions, promoting the formation of complex solid solutions [18].

One example of the capability of CCAs to reduce the critical metal content is the addition of Al in high-temperature alloys. Thus, critical metals frequently used in these alloys (Co, Ti, Mo, Nb, or W) can be partially replaced with Al. As part of the CCAs family, HEAs have received great attention in the past, especially for high-temperature applications. HEAs containing Al were the most interesting due to the strong hardening effect induced by the gradual additions of aluminum in compositions associated with transitional elements, such as Co, Cr, Fe, Ni, Ti, etc. HEAs usually form disordered structures with a very low content of intermetallic-based phases [55]. The aluminum percentage in the alloy can induce a sudden structural transformation from FCC to BCC CFC to CVC at several multi-component alloys. For example, the equimolar AlCoCrFeNiTi alloy reaches a compressive strength of 2.28 GP, an elastic modulus of 147.6 GPa, and hardness of 706 HV, all being comparable or higher than well-known superalloys [56,57]. Recent studies showed that HEAs containing Al possess improved mechanical properties at high temperatures, compared to superalloys Ni-based In 718 and Co-based Triballoy T-800 [58]. At Cr levels higher or equal to equimolar composition, HEAs have a better hardness at temperatures above 800 °C. The slow decrease in hardness of HEAs at high temperatures, as opposed to superalloys, is also remarkable.

Substitution of critical metals was also researched through CCAs in the replacement of Co with Mn in high-temperature alloys. The high content of alloying elements was replaced for the low melting temperature of Mn. Further research trials showed that the substitution of Co with Mn in the AlCoCrFeNi system generates a novel structure composed of a solid solution matrix and evenly dispersed nanometric size intermetallic compounds [59]. Mn added to CrFeCoNi improves the pitting corrosion [60] and additions of Mn to Cu containing HEAs has a beneficial influence in decreasing the element segregation, thus suppressing the galvanic corrosion [61].

Several research studies were performed for high-temperature HEAs containing refractory elements [27,28,29,30,31,32,62,63]. These alloys based on W, Mo, Nb, and Ta additions show significant high strength at room temperature and have excellent resistance to thermal softening. For example, Nb_25_Mo_25_Ta_25_W_25_ and V_20_Nb_20_Mo_20_Ta_20_W_20_ yield strength remain virtually unchanged in the range of 600–1000 °C, reaching over 400 MPa at 1600 °C [30]. These novel HEAs contain a large number of critical metals but present significantly higher properties than the conventional nickel-based alloys. In the following studies, the authors tried to reduce the refractory and critical metal content by the addition of light metals. Senkov et al. [28,31] studied low density (up to 6.57 g/cm^3^) refractory HEAs, based on the following systems: NbTiVZr, NbTiV2Zr, CrNbTiZr, and CrNbTiVZr. The alloys were obtained by the substitution of heavy elements (Mo, W, and Ta) with lighter Ti, Zr, V, and Cr. The mechanical characteristics of the resulted samples revealed comparable or even higher values than Inconel 718 and Haynes 230. Mechanical resistance of 1298 MPa at room temperature and 259 MPa at 1000 °C at high temperatures were obtained for the CrNbTiVZr system.

The addition of aluminum in high-temperature alloys was also considered beneficial for improving oxidation resistance and lower density [62,63]. Trials with different Al additions were performed in the alloys with a high Al content (AlMo_0.5_NbTa_0.5_TiZr and AlNb_1.5_Ta_0.5_Ti_1.5_Zr_0.5_) and showed remarkable strength (745 MPa and 403 MPa, respectively) at high temperatures (1000 °C), exceeding IN718 and Mar-M247 performance [32,64]. The slow diffusion characteristics of CCAs lead to superior oxidation resistance. At the same time, it has been shown that alloying refractory alloys with a high amount of Al, Cr, Ti, and Si are effective in lowering the critical metal content, obtaining an improved oxidation resistance at high temperatures [32]. Liu et al. [64] studied the effect of Al, Cr, Ti, V, and Si on the oxidation strength of CCAs with refractory properties. It is considered that all the compositions of CCAs have good oxidation resistance at temperatures up to 1300 °C.

**Figure 7:**
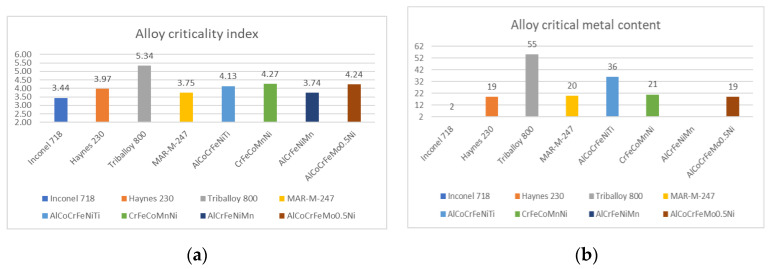
The criticality index (**a**) and the critical metal content (**b**) for the selected alloys.

Popov et al. reported that modern digital manufacturing methods such as additive manufacturing could be effective screening techniques for new chemical compositions of HEAs [65]. The specimens of Al_0.5_CrMoNbTa_0.5_ composition were additively manufactured using blended powder. The obtained results showed that multi-principal alloys could be manufactured even by in-situ alloying [65,66]. Recent research performed by Ferro et.al [67,68] elaborated an index of criticality for materials that take into consideration the abundance risk level, the sourcing and geopolitical risk, the environmental country risk, the supply risk, the economic importance, and the end-of-life recycling input rate. In order to measure the potential of CCAs for the substitution of critical metals, a criticality index was calculated for the most promising CCA compositions and compared with the conventional alloys (Figure 7). The formulas for calculation were adopted from [67,68] and the values for initial calculations from the EU2017 report [69] because some indexes were not listed in the 2020 report. The results showed that the criticality index of CCAs with comparable or better properties is less than that of conventional alloys.

The low criticality of Incaloy and Haynes alloys was not a surprise because the compositions are based mainly on Ni and Fe, but their melting temperature is relatively low (between 1200–1500 °C), and they have lower high-temperature resistance than the compared CCAs. The AlCoCrFeNiTi alloy is comparable in performance with Triballoy 800. The same can be said for the AlCrFeNiMn and Haynes 230. It is also important to mention that the listed compositions for CCAs are not the final optimal solutions, research is underway for providing industrially viable solutions.

Complex structural behavior of CCAs and recent research results in the field make these alloys a promising area of research for the substitution of critical metals.

Future research directions in the field of CCAs, from the perspective of critical raw material reduction or substitution in highly demanding applications, are based on the particular multi-component design that offers a wide variety of development possibilities. One should classify the alloys based on their structure type as predominant solid solution CCAs with none or little intermetallic content and CCAs that contain a recognizable amount of intermetallic phase and generate important mechanical properties, especially at high temperatures. Depending on critical metal nature, the prospected alloys can be chosen from one or another category. For example, Co is a solid solution former that can be replaced in the first category of alloys with an element or group of elements with similar structural effects. Si, Ti, or rare earth elements are known for the formation of hard intermetallic phases and are also good oxygen receivers, which should be replaced also by elements with similar effects.

In spite of the fact that the fabrication of CCAs is less limited compared to high-entropy and multi-principal alloys, their synthesis also requires modeling for efficient composition establishment. In order to establish viable compositions, studies need to be conducted on the selection of compatible elements or groups of elements by means of multiscale modeling and complex experimental processes, related specifically to the required properties of the material. Complex concentrated alloys, similar to high entropy alloys, develop different characteristics in the as-cast state or heat-treated state. Most of the past research has been conducted for the alloys in the as-cast state, which offered out of equilibrium and unstable structures. The multi-principal nature of CCAs requires several post-synthesis treatments to acquire the desired properties. Actually, the main benefit of multi-component alloys is the capability in tailoring properties by the manipulation of one or more elements concentration (e.g., Al, Cr, Cu, etc.).

There are still many unanswered topics in the theoretical research of CCAs. For example, it is hard to explain the solid solution hardening and sluggish diffusion effects for these types of alloys. The high concentration of the elements contributes to the confusion of which is the solvent and which one is the solute in the final structure. It is not yet known for certain that the dislocation structure and movement are influenced strongly by the lattice strains or local chemical variations. Further modeling and experimental research are needed in this very important area, toward the prediction of important properties.

Another idea on the path of replacing critical elements or alloys with CCAs is the exploration of compositional gradient design of various structural parts for certain applications. These types of structures are usually very complex but can offer interesting solutions for the material design with various properties—magnetism, electrical insulation, mechanical resistance, heat resistance, etc.

## 5. Conclusions

- The latest reports from the European Commission presented are the materials that are considered critical from the perspectives of supply risk and economic demand. Due to the increased demand from past and emerging technologies the list of critical materials increased with four more additions—bauxite, titanium, lithium, and strontium;
- There is a large number of metals among the established critical materials. The applications envisioned to be critical for the raw material supply are part of the aerospace industry, including batteries, fuel cells, wind energy, electrical motors, photovoltaics, robotics, drones, 3D printing, and digital technologies. Important metals that have become critical and are extensively used in extreme environment applications are Co, Hf, Nb, Ta, Ti, V, and W;
- Complex concentrated alloys (CCAs) represent a new family of metallic materials based on the multi-component and high proportion element concepts, similar to the previous high entropy alloys and multi-principal element alloys definitions. In this case, a lower number of elements, lower concentrations, and intermetallic compound presence are acceptable;
- Previous research in the field of the CCA families showed some preferential directions, related closely to the alloy’s improved properties—high temperature and high strength applications. There are also trials for important applications related to the critical raw materials field, namely, hydrogen storage alloys, thermoelectric materials, magnetic materials, etc.
- The main problem with the critical metals is that they are essential to related applications and are difficult to be substituted with other materials or even reduce the consumption level. In this case, a sustained effort needs to be addressed to avoid a future blockage in the supply–demand that can put at risk these vital industrial fields.
- CCAs represent a promising direction in this respect due to their multi-component nature that allows for increased flexibility in alloying combinations. Previous achievements obtained by either lowering or replacing Co in some alloys are considered promising results for the substitution of critical metal content in alloys for extreme environment applications;
- CCAs are capable of developing equal or higher characteristics for a comparable or lower criticality index.

## Figures and Tables

**Table 1:** Critical raw materials list [1].

2020 Critical Raw Materials (New as Compared to 2017 in Bold)				
Antimony	Fluorspar	Magnesium	Scandium	**Titanium**
Baryte	Gallium	Natural Graphite	Silicon	**Strontium**
Beryllium	Germanium	Natural Rubber	Tantalum	-
Bismuth	Hafnium	Niobium	Tungsten	-
Borate	Heavy Rare Earth Elements	Platinum Group Metals	Vanadium	-
Cobalt	Light Rare Earth Elements	Phosphate rock	**Bauxite**	-
Coking Coal	Indium	Phosphorus	**Lithium**	-

**Table 2:** The main complex concentrated alloys (CCA)-related definitions.

Term	Alternative Term	Abbreviation	Definition	Ref.
Multi-principal alloys	Multi-principal Element Alloys	MPA/MPEA	A composition-based definition for alloys with a large number of elements in high concentrations.	[2,6,7,8]
High-entropy Alloys	-	HEAs	Multi-component alloys with concentration of each element being between 35 and 5 at.%, based on the high entropy effect to produce single solid solution structures	[3]
Complex Concentrated Alloys	Compositionally Complex Alloys	CCAs	Alloys with a complex composition, containing a large number of elements, but not necessarily in a large proportion for each element	[4,5]

## Data Availability

As this is a review paper, no new data were generated for this paper.
